# 2841. Skipping the Urine Culture before Genitourinary Surgeries May Not be Associated with Increased Risk of Postoperative UTI

**DOI:** 10.1093/ofid/ofad500.2451

**Published:** 2023-11-27

**Authors:** Juan Teran Plasencia, William O’Brien, Lori Lerner, Marin Schweizer, Judith Strymish, Kalpana Gupta

**Affiliations:** VA Boston Healthcare System and Boston University/Department of Infectious Diseases Boston VA Healthcare System, Boston, Massachusetts; VA Boston Healthcare System, West Roxbury, MA; VA Boston Healthcare System and Boston University Chobanian & Avedisian School of Medicine/ Urology, Boston, Massachusetts; Iowa City VA Health Care System, Iowa City, Iowa; VA Boston Healthcare System, West Roxbury, MA; VA Boston Healthcare System and Boston Universiy School of Medicine, West Roxbury, MA

## Abstract

**Background:**

Detection of bacteriuria prior to most genitourinary (GU) procedures is a guideline-supported standard of care for prevention of postoperative UTI. Little is known about the frequency of concordance with guidelines and impact on outcomes. We hypothesized that GU surgeries in which a preoperative urine culture was indicated but not performed would be associated with an increased rate of postoperative UTI.

**Methods:**

We obtained national data on all elective GU surgeries performed in VHA operating rooms during 2015-2022. CPT codes were flagged as indicated for preoperative urine culture based on risk of mucosal injury and AUA guidelines. Surgeries not indicated for urine culture were excluded. We identified urine cultures performed within 45 days before surgery. UTI outcome was defined as occurrence of ICD-10 code N39.0 within 30 days after surgery.

Multiple logistic regression estimated the independent association of preoperative culture performance with the outcome of UTI. Other independent variables included age, sex, ASA Class, antibiotics, and the 29 Elixhauser comorbidities.

**Results:**

The study population included 221,018 surgeries at 123 VA Medical Centers. Mean (SD) age was 69.4 (11.0), 96.0% were men, and 76.1% were ASA Class 3-5. The most common surgeries were cystoscopy (62.8%), lithotripsy (9.9%), and TURP (8.7%). The comorbidity burden was typical for VA population studies (Table 1).

Preoperative urine cultures were performed in 65.5% of surgeries. In regression analysis controlling for comorbidities and antibiotics, the risk for postoperative UTI in those who did not have a urine culture performed despite being indicated, compared with those who did, was OR 0.56 [95%CI 0.53-0.58] (Table 2).

**Figure:**
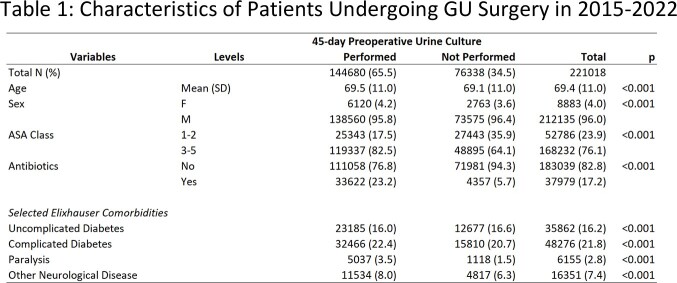

Multivariate Logistic Regression to Predict 30-day Postoperative UTI in GU Surgery Patients

**Figure:**
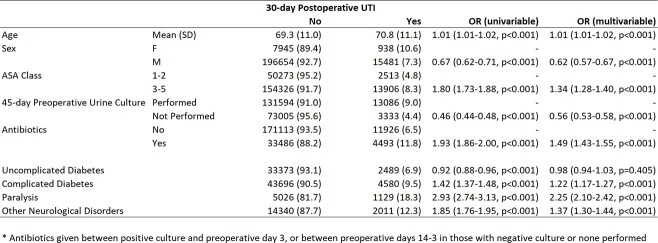

**Conclusion:**

In this large national cohort covering 8 years of GU surgeries, we found that non-performance of an indicated urine culture was common but was not independently associated with increased risk of postoperative UTI. The finding of significantly reduced risk was surprising and suggests that confounding patient factors, rather than procedure type, are driving the risk of postoperative UTI. Further work is warranted to identify these factors and explore the potential for de-implementation of standardized preoperative urine cultures for lower risk GU surgery patients.

**Disclosures:**

**Lori Lerner, MD**, Janssen Pharmaceutical: Grant/Research Support **Kalpana Gupta, MD, MPH**, GSK: Advisor/Consultant|IDSA UTI Guidelines: Advisor/Consultant|qiagen: Advisor/Consultant|UpToDate: Advisor/Consultant

